# Circulation of fluconazole-resistant *Candida auris* in Peru confirmed by genomic analysis

**DOI:** 10.1590/0074-02760260027

**Published:** 2026-07-31

**Authors:** Rolando Paredes-Gago, Alicia Nuñez-Llanos, Clelia Cespedes-Roman, Vanessa Izarra-Rojas, Veronica Hurtado, Carlos Padilla-Rojas, Omar Caceres-Rey, Wendy Lizarraga

**Affiliations:** 1 National Centre for Public Health, Instituto Nacional de Salud, National Reference Laboratory of Mycology, Lima, Peru; 2 National Centre for Public Health, Instituto Nacional de Salud, Innovation and Development Area, Lima, Peru

**Keywords:** Candida auris, genomic surveillance, antifungal resistance, fluconazole, phylogeny

## Abstract

**BACKGROUND:**

*Candida auris* is an emerging fungal pathogen associated with invasive infections, notable for nosocomial spread and multidrug resistance. Despite its public health importance, genetic data from Peruvian isolates remain limited.

**OBJECTIVES:**

To perform a multi-isolate genomic analysis of Peruvian *C. auris* isolates, including epidemiological analysis, clade assignment, phylogenetic reconstruction, and characterisation of antifungal resistance-associated mutations.

**METHODS:**

Twenty clinical samples from hospitals in Lima and Callao were identified using MALDI-TOF and subjected to antifungal susceptibility testing. Genomic DNA was sequenced, and genomes meeting quality criteria (n = 19) were included in the analysis. Phylogenomic and *ERG11* phylogenetic analyses were performed, and protein modelling combined with molecular docking was used to assess interactions with lanosterol 14-α-demethylase.

**FINDINGS:**

All analysed isolates belonged to clade IV and exhibited resistance to fluconazole. The *ERG11* gene harboured four relevant mutations; notably, K143R was located within the hemi-binding region of lanosterol 14-α-demethylase and was predicted, based on molecular docking, to potentially alter fluconazole binding. These findings highlight the circulation of a resistant clade and underscore the need for strengthened genomic surveillance and antifungal stewardship strategies.

**MAIN CONCLUSIONS:**

This study expands the genomic data available for *C. auris* and reinforces the urgent need for sustained genomic surveillance to monitor and contain antifungal resistance.

*Candida auris* is an emerging fungal pathogen of major public health concern due to its multidrug resistance and its ability to cause outbreaks in hospitals and other healthcare facilities. Since its first identification in Japan in 2009,^1^ *C. auris* has shown high resistance to commonly used antifungals and a remarkable capacity to persist in healthcare environments, underscoring the need for active genomic surveillance to monitor its spread and evolution.^2^ ^,^ ^3^

In February 2021, the Pan American Health Organization (PAHO) issued an epidemiological alert reporting an increase in *C. auris* outbreaks in healthcare settings during the Coronavirus disease 19 (COVID-19) pandemic. More recently, the World Health Organization (WHO) included this pathogen in the fungal priority pathogens list because of its global dissemination, nosocomial transmission, and clinical impact.^4^ ^,^ ^5^

Phylogenetic studies based on whole-genome sequencing (WGS) and single nucleotide variants (SNPs) have described six geographically distinct clades of *C. auris*: Clade I (South Asia, mainly India and Pakistan), Clade II (East Asia, primarily detected in Japan and South Korea), Clade III (Africa, predominant in South Africa and other African regions), and Clade IV (South America, mainly reported in Venezuela and Colombia). Clade V has been described based on a limited number of isolates from Iran, while Clade VI is a recently proposed lineage identified in Bangladesh and Singapore.^6^ ^,^ ^7^ ^,^ ^8^ ^,^ ^9^

The *C. auris* genome is approximately 12.3-12.5 Mb across seven chromosomes. Its genomic architecture includes an expanded repertoire of genes linked to antifungal resistance (*e.g.*, efflux pumps and drug-modifying enzymes) and mutations in key resistance genes such as *ERG11* (azoles) and *FKS1* (echinocandins). Genomic variability among clades, including chromosomal rearrangements and mobile elements, has been associated with differences in resistance mechanisms.^10^ ^,^ ^11^ ^,^ ^12^ ^,^ ^13^

Information on sequenced genomes of *C. auris* in Peru remains limited. A literature search identified only two genomic reports. The first described the genomic sequencing of a *C. auris* isolate obtained from an intensive care unit (ICU) patient, showing that this strain belongs to clade IV and shares 98-99% similarity with isolates from Venezuela and Colombia.^14^ A more recent study reported the first draft genome of *C. auris* in Peru, identifying its classification within clade IV and the presence of the *ERG11* (K143R) mutation, which is associated with fluconazole resistance.^15^ However, comprehensive studies addressing genomic diversity, phylogenetic relationships, and antifungal resistance mechanisms across multiple isolates in Peru remain lacking.

Genomic surveillance using WGS and SNP analyses is essential for outbreak investigation, early detection, and monitoring pathogen evolution in healthcare settings. In this context, we report the first multi-isolate genomic analysis of *C. auris* recovered from hospitals in Lima and Callao, Peru, expanding upon previous reports based on single-isolate genomes.

## MATERIALS AND METHODS

*Clinical and epidemiological data* - This study was a retrospective observational analysis of *C. auris* isolates collected between 2020 and 2024 through the national surveillance system. The inclusion criteria were isolates confirmed as *C. auris* by matrix-assisted laser desorption/ionisation time-of-flight mass spectrometry (MALDI-TOF MS) and available for antifungal susceptibility testing and genomic sequencing. A total of 20 clinical isolates from hospitals in Lima and Callao were included, representing all confirmed cases received by the Laboratory Response Network (LRN) of Mycology during the study period.

Each sample was accompanied by clinical-epidemiological records from which relevant data were obtained. These data were systematically reviewed to identify variables that allowed characterisation of the patient profile. All samples were stored at room temperature using the Castellani method.^16^ The isolates were obtained during routine diagnostic activities and antifungal resistance surveillance in yeast-like fungi. No personal data or information that could enable patient identification were accessed.

*Phenotypic identification* - Protein extraction of yeast-like fungal isolates was performed for analysis by MALDI-TOF MS, following the yeast protocol of the MALDI Biotyper system (Bruker Daltonics, Germany). Spectra were acquired using FlexControl v3.4 (build 85.1), species identification was performed using the BDAL (Bruker Daltonic Library) database.^17^ Initially, isolates were incubated in 3 mL of Sabouraud broth (Liofilchem) for 24 h at 37ºC. Subsequently, 1.5 mL of culture was collected and centrifuged at 13,000 rpm for 2 min. The resulting pellet was washed with HPLC-grade water (Supelco) under agitation, followed by a second wash with absolute ethanol (Applichem). After drying the sediment at 37ºC for 10-15 min, another centrifugation was done. 1 μL of the supernatant was applied onto the MALDI target plate, formic acid (Millipore) and acetonitrile (Supelco) were added to facilitate protein release, followed by 1 μL of HCCA matrix (Bruker), and then analysed in the instrument.

*Antifungal susceptibility testing* - Antifungal susceptibility testing was performed using the Sensititre YeastOne kit (Thermo Fisher Scientific), based on the colorimetric broth microdilution method, following the recommendations of the Clinical and Laboratory Standards Institute (CLSI). *Candida krusei* ATCC 6258 was used as the quality control strain. A 0.5 McFarland suspension of each isolate was prepared in demineralised water, inoculated into YeastOne broth (Thermo Fisher Scientific), and transferred to YeastOne YO10 antifungal plates (Thermo Fisher Scientific). Plates were incubated at 35ºC for 24 h, and minimum inhibitory concentrations (MICs) were determined using the Vizion reading system (Thermo Fisher Scientific), according to the manufacturer’s instructions. Susceptibility interpretation was performed based on the breakpoints proposed by the Centres for Disease Control and Prevention (CDC).^18^

*DNA extraction* - Total genomic DNA was extracted from cultures of clinical *C. auris* isolates previously identified by MALDI-TOF MS. Approximately 50 mg of biomass was collected from a pure fungal culture grown for 24 h on Sabouraud agar (Liofilchem) and aseptically transferred into a 1.5 mL microcentrifuge tube. The samples were subjected to controlled freezing at -40ºC for 48 h. Subsequently, cell lysis and DNA extraction were performed using the PureLink™ Genomic DNA Mini Kit (Thermo Fisher Scientific, USA), following the manufacturer’s instructions. The DNA concentrations were determined using a Qubit 4.0 fluorometer (Thermo Fisher Scientific, USA) and NanoDrop 2000c spectrophotometer (Thermo Fisher Scientific, USA). Samples selected for sequencing had DNA concentration greater than 10 ng/μL and a mean 260/280 nm absorbance ratio of 1.92.

*Library preparation and sequencing* - Genomic DNA libraries were constructed with a Nextera XT DNA Library Preparation Kit (Illumina, USA), according to the manufacturer’s protocol. The libraries were subsequently sequenced on the Illumina MiSeq platform using the MiSeq Reagent Kit v3 (Illumina, USA) with 250 bp paired-end reads for 600 cycles.

*Genome assembly and annotation* - Raw read quality was evaluated using FastQC v0.12.1.^19^ Raw reads were adapter- and quality-trimmed using Trimmomatic v0.9.^20^ High-quality reads were taxonomically classified using Kraken v2.1.3^21^ with the PlusPF database. *C. auris* genomes were de novo assembled using SPAdes v3.13.1.^22^ The contigs were further polished with Pilon v1.12^23^ to correct nucleotide errors, including artificial SNPs and indels. Genome scaffolding and improvement were conducted using RagTag v2.1.0.^24^ Repetitive elements were predicted using RepeatModeler2 v2.0.4^25^ with the LTRStruct parameter, followed by masking with RepeatMasker v4.1.7.^26^ Gene prediction was performed using the Companion server v2.2.11,^27^ employing *C. auris* strain B8441 (5,584 genes) as the reference genome. BUSCO v5.8.2.^28^ was used to verify the completeness of the genomes against the *saccharomycetes_odb*12 (2319 single-copy orthologs) database.

*Phylogenomic analysis* - Orthogroup identification and phylogenomic reconstruction were performed using OrthoFinder v3.1.0.^29^ First, 25 complete *C. auris* genomes were downloaded from the NCBI database, and their corresponding protein sequences were obtained using Companion. Clade identification was conducted through a bibliographic search of each isolate in the PubMed database. The resulting phylogenomic tree was edited and visualized using FigTree (http://tree.bio.ed.ac.uk/software/figtree/) and the Interactive Tree Of Life (iTOL) web resource (https://itol.embl.de/).

*Phylogenetic analysis of the ERG11 gene* - A BLASTn search was performed using the nucleotide sequence of the *ERG11* gene to identify sequences with the highest similarity across different strains. The retrieved sequences were aligned using MAFFT,^30^ and subsequently translated into amino acid sequences. A phylogenetic tree was then constructed based on this amino acid alignment using IQ-TREE2,^31^ with ModelFinder identifying LG+G4 as the best-fitting substitution model. The resulting phylogenetic tree was visualized using FigTree and Microreact (https://microreact.org/) and subsequently edited in Inkscape (https://inkscape.org/).

*Molecular docking* - To obtain the best protein model, homology modelling was performed using SWISS-MODEL,^32^ and fold-recognition modelling was conducted with Phyre2 v2.2.^33^ The seven generated models were evaluated using the ModFold8 server^34^ and PDBsum,^35^ analysing Ramachandran plots, confidence scores, p-values, and the global model quality score. Subsequently, molecular dynamics simulations of the best model were performed with GROningen MAchine for Chemical Simulations (GROMACS).^36^ Molecular docking was conducted using AutoDock Vina, and structural visualisation was carried out with PyMOL. Protein-ligand interaction analysis was performed using LigPlot.

*Ethical considerations* - This study was conducted using data obtained from routine laboratory surveillance activities rather than a specifically designed research project. Therefore, according to institutional policies and national regulations, ethical review and approval were not required. All data were anonymised and handled in accordance with the principles of the Declaration of Helsinki.

## RESULTS

*Clinical and epidemiological data* - Of the 20 samples analysed, 95% of cases were concentrated in the district of Lima, suggesting a higher prevalence of *C. auris* in this region. Only one case was reported in the Callao region. Regarding temporal distribution, one sample (5%) was collected in 2024, 50% in 2022, 25% in 2023, and the remaining 20% (four samples) between 2020 and 2021. A higher proportion of isolates was observed in male patients (70%). The most affected age group was middle-aged adults, with a mean age of 49 years (Table I). Isolated cases observed among both adolescent and geriatric populations of both sexes highlight the ability of the pathogen to affect immunocompromised or vulnerable groups.

**TABLE I:** Clinical and epidemiological characteristics of 20 *Candida auris* cases from Lima and Callao (2020-2024)

Isolate ID	Year of isolation	Location	Sample type	Sex	Age (years)	Comorbidities	Clinical characteristics	Hospital ward
CA1PER	2020	Lima	Blood	M	67	Diabetes	COVID-19 pneumonia, antimicrobial use	Hospital ward
CA2PER	2020	Lima	Blood	M	44	Diabetes	Antimicrobial use	Hospital ward
CA3PER	2021	Lima	Blood	F	59	Rheumatoid arthritis	Corticosteroid therapy, antimicrobial use	Hospital ward
CA4PER	2021	Lima	Blood	M	61	NR	COVID-19 pneumonia, antimicrobial use	ICU
CA5PER	2022	Lima	Blood	M	16	Tuberculosis	NR	ICU
CA6PER	2022	Lima	Rectal swab	M	77	Renal failure	NR	Hospital ward
CA7PER	2022	Lima	Blood	F	25	NR	Abdominal surgery, antimicrobial use	Hospital ward
CA8PER	2022	Lima	Blood	M	27	Diabetes	Endocarditis, antimicrobial use	Hospital ward
CA9PER	2022	Lima	Rectal swab	M	56	NR	NR	Hospital ward
CA10PER	2022	Lima	Rectal swab	M	16	NR	Gastrostomy patient	ICU
CA11PER	2022	Lima	Rectal swab	M	88	Renal failure	NR	ICU
CA12PER	2022	Lima	Rectal swab	F	71	NR	Gastrostomy patient	ICU
CA13PER	2022	Lima	Tissue	F	15	Renal failure	Antimicrobial use	Hospital ward
CA14PER	2022	Lima	Blood	M	37	Tuberculosis	COVID-19 pneumonia, antimicrobial use	Hospital ward
CA15PER	2023	Lima	Blood	M	27	NR	NR	Hospital ward
CA16PER	2023	Lima	Blood	M	26	Systemic lupus erythematosus	Antimicrobial use	Hospital ward
CA17PER	2023	Lima	Blood	F	79	NR	NR	Hospital ward
CA18PER	2023	Lima	Blood	M	50	NR	Antimicrobial use	Hospital ward
CA19PER	2023	Lima	Blood	F	79	NR	NR	Hospital ward
CA20PER	2024	Callao	Tissue	M	54	NR	NR	Hospital ward

NR: not reported; ICU: intensive care unit.

The most frequent clinical histories among patients included prior broad-spectrum antimicrobial use were reported in 50% (10/20) of the cases, followed by COVID-19-associated pneumonia, prolonged corticosteroid use, and a history of surgery. Some patients received antifungal therapy, with caspofungin and fluconazole being the most used agents, and some cases were also treated with amphotericin B and anidulafungin. However, antifungal treatment was not consistently recorded across all patients.

Regarding comorbidities, diabetes mellitus was present in 15% (3/20) of patients; chronic renal failure, tuberculosis, and autoimmune diseases such as systemic lupus erythematosus and rheumatoid arthritis were identified. Specific clinical conditions, including patients with gastrostomy tubes, were also documented, reflecting states of prolonged medical dependence.

Of the total *C. auris* isolates sequenced, 75% were isolated from patients hospitalised in general wards, while 25% were isolated from patients admitted to ICUs.

*Phenotypic description: identification of C. auris* - Identification of the 20 isolates was performed using MALDI-TOF MS. The generated spectra showed a set of specific and reproducible peaks within the mass range of 2,000 to 20,000 Da. All 20 analysed isolates displayed intense and consistent peaks between 5,500 and 8,000 Da, which facilitated their identification as *C. auris* through comparison with the MALDI Biotyper database (Bruker).

This identification was based on the species-specific protein profile and yielded score values higher than 2.0 in all cases, indicating reliable and accurate identification.

**Fig. 1:**
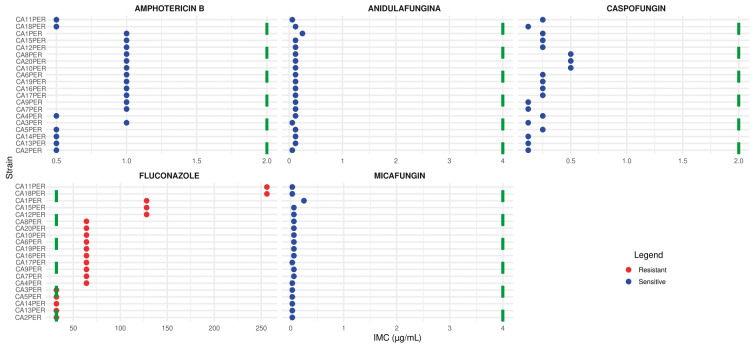
antifungal susceptibility profile of the 20 *Candida auris* isolates against amphotericin B, anidulafungin, caspofungin, fluconazole, and micafungin. Each data point represents an individual isolate, and green horizontal lines indicate the clinical breakpoints proposed by the Centres for Disease Control and Prevention (CDC) for resistance interpretation.

*Antifungal susceptibility* - Antifungal susceptibility testing revealed that 100% of the isolates were resistant to fluconazole and susceptible to amphotericin B, anidulafungin, caspofungin, and micafungin (Fig. 1). This pattern remained consistent throughout the five-year study period, with no variations observed in resistance or susceptibility profiles over time.

For fluconazole, 100% of the isolates exhibited MIC values equal to or greater than 32 µg/mL, exceeding the resistance breakpoint (≥ 32 µg/mL), indicating a fluconazole-resistant profile. In contrast, the MIC values obtained for amphotericin B (≤ 1 µg/mL), anidulafungin, micafungin, and caspofungin (≤ 0.5 µg/mL) were consistently below their respective breakpoints (amphotericin B ≥ 2 µg/mL; echinocandins ≥ 4 µg/mL or ≥ 2 µg/mL, depending on the antifungal), indicating *in vitro* susceptibility to these four antifungal agents in all evaluated isolates.

Additionally, MIC values for posaconazole ranged from 0.015 to 0.12 µg/mL, while those for voriconazole ranged from 0.12 to 0.5 µg/mL. No elevated MICs were observed for any of these antifungals; however, it was not possible to perform a categorical classification of susceptibility or resistance due to the lack of defined clinical breakpoints.

No relevant differences in MIC distributions were observed between isolates obtained from colonization sources (rectal swabs) and those from invasive infections (blood and tissue samples), as values remained within comparable ranges across all antifungal agents.

Detailed MIC distributions for all isolates and antifungal agents are provided in Supplementary data (Table).

*Genomic analysis: de novo assembly* - The *C. auris* genomes obtained had an average size of 12.39 Mb and had between 39 and 74 contigs. These genomes were assembled with 98.29% coverage relative to the reference genome, had an average depth of 203.49x, and a GC content of 45.14%. Overall, these results indicate that the assembled genomes are complete, well structured, and highly similar to the reference genome *C. auris* B11245 (Table II).

**TABLE II:** Assembly and annotation metrics of the 20 assembled genomes

Strain	Total length (bp)	# contigs (> 200 bp)	Genome (%)	Coverage	GC (%)	Number of genes
CA1PER	12,366,764	56	98.42	202.672x	45.14	5,588
CA2PER	12,359,436	41	98.444	153.378x	45.15	5,581
CA3PER	12,369,349	48	98.418	186.516x	45.14	5,588
CA4PER	12,372,834	39	98.405	135.981x	45.16	5,588
CA5PER	12,355,246	55	98.224	150.642x	45.18	5,577
CA6PER	12,363,351	56	98.406	148.129x	45.15	5,576
CA7PER	12,366,966	53	98.351	128.839x	45.16	5,582
CA8PER	12,376,544	46	98.411	115.605x	45.15	5,589
CA9PER	12,374,305	68	98.437	338.108x	45.14	5,583
CA10PER	12,371,467	53	98.436	217.944x	45.16	5,580
CA11PER	12,372,053	55	98.434	218.278x	45.15	5,584
CA12PER	12,366,041	54	98.423	225.184x	45.15	5,583
CA13PER	12,367,696	70	98.423	223.328x	45.15	5,578
CA14PER	12,369,758	58	98.394	176.033x	45.16	5,574
CA15PER	12,357,760	62	98.46	298.765x	45.14	5,582
CA16PER	12,373,295	47	98.465	298.765x	45.15	5,577
CA17PER	12,357,138	36	98.398	146.984x	45.17	5,581
CA18PER	12,326,928	56	98.253	667.951x	45.13	5,570
CA19PER	12,572,015	72	98.355	25.8591x	44.93	5,584
CA20PER	12,624,799	72	96.261	10.8669x	45.09	4,715

GC: percentage of guanine and cytosine.

The analysis showed that all genomes contained the same number of orthologous genes (2,319), which were classified as complete, single-copy, duplicated, fragmented, or missing [Supplementary data (Figure)]. Of the total orthologous genes identified, more than 99.18% were complete, and only a minimal proportion were missing. These findings demonstrate that the genomes are of high quality with low levels of fragmentation.

*Genome annotation* - The *C. auris* genomes had an average of 5,538 annotated genes, with a mean gene density of 433.12 genes per megabase (genes/Mb). Of the total genes identified, 96.4% corresponded to protein-coding genes. Additionally, an average of 494 genes contained multiple coding sequences (CDS).

Table II presents a complete description of each annotated genome. Strain CA20PER was excluded from further analyses due to its low coverage (10.87x). Prior to deposition in NCBI, we filtered the genomes of the 20 strains included in the analysis using a minimum sequence length threshold of > 200 bp. The data are available under BioProject ID PRJNA1389625.

*Phylogenomic analysis* - From the analysis of orthologous gene groups across 44 genomes, a total of 244,370 genes were identified. Of these, 244,073 genes (99.9%) were assigned to orthogroups, while 297 genes could not be grouped. In total, 5,639 orthogroups were detected, encompassing 99.9% of the identified genes. Among them, only six orthogroups were classified as specific, comprising 16 genes. Additionally, 4,869 orthogroups corresponded to single-copy genes.

**Fig. 2:**
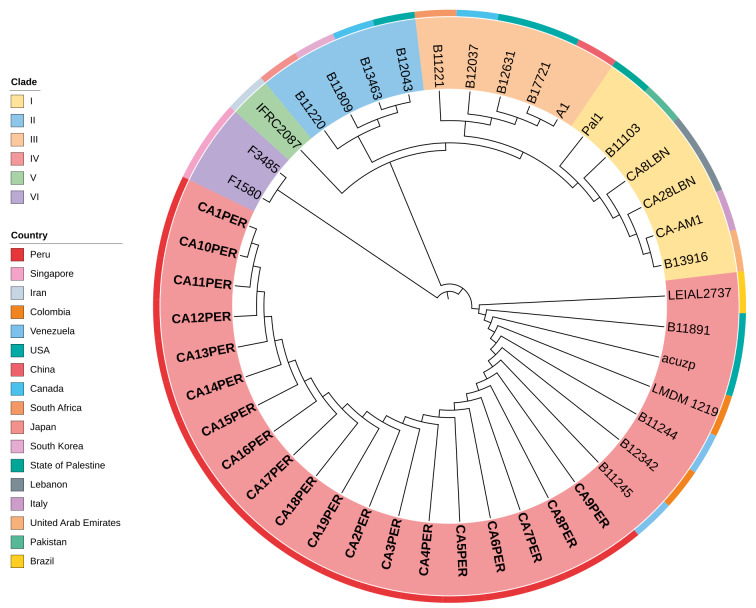
phylogenomic analysis of 44 *Candida auris* genomes. The tree depicts the six phylogenetic clades (I-VI) and the origin country of each isolate. The 19 Peruvian *C. auris* strains are highlighted in bold, all belonging to clade IV.

The resulting phylogenomic tree is shown in Fig. 2, in which the 19 *C. auris* strains (CA1PER-CA19PER) cluster within clade IV, primarily grouping with isolates from Colombia, Venezuela, the United States, and Brazil. The analysis indicates that only clades II, III, and IV are circulating in the Americas, whereas clades I, V, and VI are mainly distributed across Europe, Asia, and Africa.

**Fig. 3:**
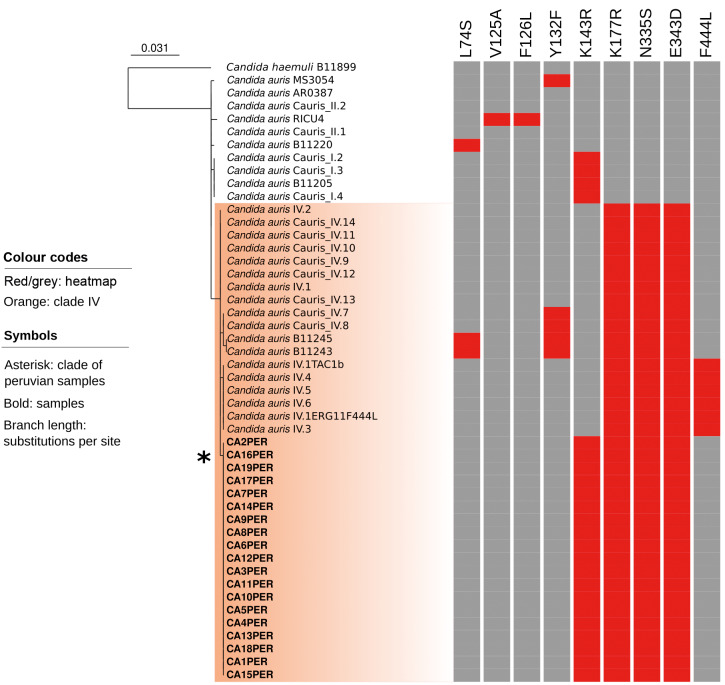
phylogenetic analysis of the *ERG11* gene analysis of 19 *Candida auris* isolates. The graph highlights clade IV strains in orange shading. The 19 Peruvian isolates are shown in bold and marked with an asterisk (*). The heatmap on the right indicates the presence (red) or absence (grey) of mutations in the *ERG11* gene.

*Phylogenetic analysis of the ERG11 gene* - All strains harboured mutations associated with fluconazole resistance in the *ERG11* gene, which encodes the enzyme 14-α-sterol demethylase, a key component in ergosterol biosynthesis and a primary target of azole antifungals. All 19 strains carried the nucleotide substitution A428G, which resulted in the amino acid change K143R. Additionally, substitutions K177R, N335S, and E343D were present in all isolates. A detailed depiction of these mutations is shown in Fig. 3.

*Molecular docking* - The best model of the Lanosterol 14-α-demethylase protein was obtained using homology modelling with C4Y9P2.1.A as a template, a *Candida lusitaniae* model with an identity of 80.50% and a GMQE value of 0.95. The best model showed 91.8% of amino acids in the most favourable zone, a CERT of 3.088E-6 and a Global model quality score of 0.7212.

**Fig. 4:**
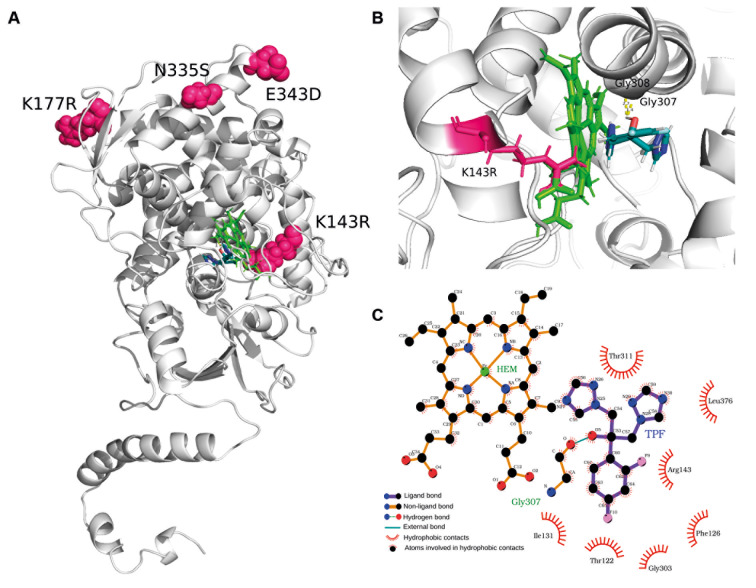
structural modelling of amino acid substitutions in Lanosterol 14-α-sterol demethylase (CYP51) from strain *Candida auris* CA1PER. (A) Modeled CYP51 protein highlighting the amino acid mutation in fuchsia. The heme group (HEM) is shown in green and fluconazole (TPF) in turquoise. (B) Close-up view of molecular docking (rotated 180°) showing the interaction between K143R and the heme group, and residues interacting with TPF within 4 Å. (C) Graphical representation of the molecular interaction between TPF and CYP51.

In the protein simulation of Lanosterol 14-α-demethylase (Fig. 4A), among the four mutations identified, only K143R interacted directly with the heme group, which serves as an intermediary between the protein and fluconazole. Fig. 4B shows fluconazole interacting with residues Gly307 and Gly308. Additionally, Fig. 4C illustrates interactions involving Gly307, Thr311, Leu376, Arg143, Phe126, Gly303, Thr122, and Ile131.

## DISCUSSION

*Candida auris* is a pathogen of major public health concern, classified by the WHO and the CDC as an urgent threat due to its multidrug resistance and capacity to cause nosocomial outbreaks. In Peru, cases have been primarily associated with healthcare-associated infections, with circulating strains showing resistance to fluconazole.^14^ In 2020, the Peruvian Ministry of Health issued epidemiological alert AE-027-2020, mandating notification of *C. auris* infections and recommending confirmation using screening tests and MALDI-TOF MS at the National Institute of Health (NIH). This alert also establishes the reporting of confirmed cases and suspected outbreaks within 24 h through the national surveillance system; however, *C. auris* is currently not included as a formally mandatory notifiable disease under a specific national regulation, but rather managed within the framework of healthcare-associated infection surveillance. This study represents the first multi-isolate genomic analysis of *C. auris* isolates from Peru, aiming to elucidate their epidemiology, genomic diversity, and resistance mechanisms.

Ninety-five percent of the confirmed *C. auris* cases originated from healthcare facilities in Lima within the framework of a passive surveillance system. Although suspected isolates were received from other regions, none were confirmed as *C. auris*. This concentration likely reflects greater diagnostic capacity and detection in highly complex metropolitan hospitals and the inherent difficulties in identifying this pathogen using conventional methods, which may contribute to its underreporting.^37^ Furthermore, challenges in accurate identification using conventional techniques may lead to misclassification or confusion with other *Candida* species.^38^

The temporal distribution, with most samples collected between 2022 and 2023, aligns with the global trend of increasing cases following the COVID-19 pandemic, a period during which intensive antimicrobial use, mechanical ventilation, and prolonged hospital stays favoured the emergence of resistant strains. The predominance of cases in male patients (70%) and a mean age of 49 years describe the demographic profile of the studied cohort. This profile is consistent with populations in which comorbidities such as diabetes mellitus and chronic kidney disease are frequent, both of which are widely recognised risk factors for infection by *C. auris* and other non-*albicans Candida* species.^39^ ^,^ ^40^

Regarding antifungal treatment, the frequent use of caspofungin and fluconazole, together with the fact that a significant proportion of patients had no recorded antifungal therapy, highlights gaps in therapeutic management and the challenge of multidrug resistance, particularly given reports that some *C. auris* strains already exhibit mechanisms of evasion against amphotericin B, azoles, and echinocandins.^41^

On the other hand, the higher detection of isolates in general hospital wards (75%) compared to ICUs (25%) indicates that surveillance should extend beyond critical care settings and supports the need for comprehensive infection control programs across all hospital areas.

Accurate identification of *C. auris* remains a challenge in clinical mycology due to its phenotypic similarity to other species within the *C. haemulonii* complex and to other *Candida* species when using conventional and semi-automated methods. In this study, all 20 evaluated isolates were correctly identified using MALDI-TOF MS, obtaining score values above 2.0 and intense, reproducible protein peaks between 5,500 and 8,000 Da. Our findings are consistent with previous reports demonstrating the stability and specificity of the *C. auris* protein profile,^42^ in which high-resolution mass spectrometry (LC-Orbitrap) achieved a correct identification rate of 99.6%, with detected peaks ranging between 5,000 and 9,000 Da, closely matching those observed in the present study.

Likewise, the consistently high scores obtained for all 20 isolates support the adequate performance of the database used in this study.^43^ These authors demonstrated that updating spectral libraries with reference spectra from all five genetic clades enables log score values greater than 2.0 for all evaluated strains and prevents misidentifications associated with incomplete databases that lack spectra from *C. auris* isolates originating from different geographic regions. Such limitations may otherwise result in low scores or confusion with phylogenetically related species.

Resistance analysis showed that 100% of the *C. auris* isolates were resistant to fluconazole (MIC ≥ 32 µg/mL), whereas all strains remained susceptible to amphotericin B and echinocandins (MICs ≤ 1 µg/mL and ≤ 0.5 µg/mL, respectively). This pattern, which was consistent over the five-year study period, reflects a widespread and stable resistance to fluconazole, a feature well-documented for *C. auris* globally. This situation is concerning because it limits first-line therapeutic options and increases reliance on alternative antifungals such as echinocandins and amphotericin B, which may, in turn, facilitate the future emergence of additional resistance given the pathogen’s capacity for multidrug resistance.

Several international studies corroborate the high levels of fluconazole resistance in *C. auris*. In India, multicentre investigations involving more than 300 isolates reported approximately 90% resistance to fluconazole, alongside low resistance to amphotericin B (8%) and echinocandins (< 2%).^44^ Similar findings have been observed in Europe, with resistance rates ranging from 87% to 100% for fluconazole and very low resistance rates for echinocandins.^45^ In South America, the situation is more heterogeneous: an outbreak in Brazil showed low resistance to all antifungals tested,^46^ whereas in Colombia, moderate levels of resistance were reported, with 35-37% resistance to fluconazole and 21-33% resistance to amphotericin B.^47^

Although *C. auris* represents a major public health problem, the number of complete genomes available in the NCBI database remains limited (57 genomes), most of which originate from North America and Europe. This underrepresentation hinders genomic surveillance in South America and underscores the need to expand regional genomic data to better understand antifungal resistance mechanisms and monitor their evolution.

While a recent report described a draft genome from a single Peruvian *C. auris* isolate,^15^ comprehensive genomic analyses across multiple isolates are still lacking. Here, we present the first multi-isolate genomic study of *C. auris* in Peru, including 20 clinical strains. In this context, 19 Peruvian genomes were successfully obtained with 98.29% coverage and an average sequencing depth of 203.49x. The genomes had an average size of 12.39 Mb, a GC content of 45.14%, and approximately 5,538 genes, which is consistent with the reported genome size range of 12.1-12.7 Mb and an estimated gene content of around 5,500 genes.^15^ ^,^ ^48^

At the genomic level, we observed that the Peruvian isolates belong to clade IV. This has also been reported in other previously described Peruvian genomes of *C. auris*.^14^ ^,^ ^15^ The *C. auris* isolates in this study are more closely related to strains from Colombia, Venezuela, the United States, and Brazil, in agreement with the previously reported geographic distribution of this South American clade.^14^ ^,^ ^49^ Although clades II and III have also been reported circulating in the Americas, these clades were not detected in the present study. The coexistence of multiple clades in certain regions worldwide has been associated with increased genetic diversity and the potential emergence of novel lineages.^50^

The analysed strains exhibited phenotypic resistance to fluconazole corroborated at the genomic level by mutations in the *ERG11* gene associated with resistance. The *ERG11* gene encodes the enzyme 14-α-sterol demethylase (CYP51) involved in ergosterol biosynthesis, a key structural component of the fungal cell membrane. Due to its biological importance, 14-α-sterol demethylase is the primary target of azole antifungals such as fluconazole.^51^

Across the 19 evaluated strains, we observed an identical resistance profile consisting of four mutations in the *ERG11* gene. Among these, only K143R appears to play a major role in resistance, owing to its interaction with the heme group involved in fluconazole binding. These findings are consistent with previous studies that have reported K143R as a mutation associated with fluconazole resistance,^52^ and it has also been recently identified in the *C. auris* 8H Peruvian isolate.^15^ Importantly, phylogenetic analysis of the *ERG11* gene in clade IV demonstrates that the K143R mutation was present in all 19 strains analysed in this study. Our molecular docking analyses indicate that the mutations K177R, N335S, and E343D are located in regions distant from the fluconazole-binding active site; these results suggest that they represent clade-specific genetic variants rather than determinants of fluconazole resistance as previously was proposed.^52^ These same three ERG11 substitutions have been reported in isolates from Latin America (Colombia and Panama), in contrast to isolates from India, New York/New Jersey, and Germany.^53^ ^,^ ^54^ Also, our molecular dicking analysis suggests that the amino acid interaction profile is composed of the residues Gly307, Thr311, Leu376, Arg143, Phe126, Gly303, Thr122, and Ile131 because these interact with fluconazole. These observations align with findings from a previous study conducted on a Mexican isolate.^55^

This study represents the first multi-isolate genomic analysis of *C. auris* in the national territory. However, the findings are subject to a limited sample size (n = 19), which restricts the characterisation of greater intraclade genomic diversity or the identification of less prevalent phylogenetic lineages in the region.

Furthermore, the origin of 95% of the isolates from the Lima metropolitan region introduces an intrinsic detection bias, linked to the diagnostic infrastructure of high-complexity hospital centres in the capital. Consequently, these results may not reflect the comprehensive epidemiological dynamics across other regions of the country, where underreporting is likely due to technical barriers in the conventional microbiological identification of this emerging pathogen.

Additionally, given that the evidence presented derives from a passive surveillance system and routine analytical activities, it is imperative to implement prospective case-finding protocols and strengthen the genomic surveillance network at the national level. This will allow for a comprehensive understanding of the geographic dispersal and evolutionary heterogeneity of *C. auris* in Peru.

Finally, our study integrates phenotypic and genomic characterization of *C. auris*, providing novel insights into the genomic diversity and fluconazole resistance mechanisms of this emerging pathogen in Peru. Given its nosocomial transmission and the emergence of multidrug-resistant strains,^56^ it is essential to strengthen integrated surveillance — both phenotypic and genomic — to better elucidate the molecular mechanisms underlying antifungal resistance and to support the implementation of effective mitigation strategies and action plans in response to its increasing spread.

## SUPPLEMENTARY MATERIALS

Supplementary material

## Data Availability

The genome sequences used for phylogenomic, and phylogenetic analyses are available in the NCBI GenBank database with the accession numbers provided in the manuscript. Phylogenomic analysis: *C. auris* F3485 (GCA_032715285.1), *C. auris* F1580 (GCA_032714025.1), *C. auris* IFRC2087 (GCA_016809505.1), *C. auris* LMDM 1219 (GCA_041381755.1), *C. auris* B11245 (GCA_008275145.1), *C. auris* B12342 (GCA_016772155.1), *C. auris* B11891 (GCA_047655445.1), *C. auris* B11244 (GCA_031357835.2), *C. auris* B17721 (GCA_016772175.1), *C. auris* B12631 (GCA_016772195.1), *C. auris* A1 (GCA_014217455.1), *C. auris* B12037 (GCA_016772215.1), *C. auris* B11221 (GCA_031357565.2), *C. auris* B11220 (GCF_003013715.1), *C. auris* B12043 (GCA_016495645.1), *C. auris* B13463 (GCA_016495665.1), *C. auris* B11809 (GCA_016495685.1), *C. auris* Pal1 (GCA_046563085.1), *C. auris* CA28LBN (GCA_019039315.1), *C. auris* CA8LBN (GCA_019039635.1), *C. auris* CA-AM1 (GCA_014673535.1), *C. auris* B13916 (GCA_016772235.1), *C. auris* B11103 (GCA_031359945.2), *C. auris* LEIAL2737 (GCA_047910675.1), *C. auris* acuzp (GCA_047655325.1). Phylogenetic analysis: *C. auris* AR0387 (MK294633.1), *C. auris* Cauris_II.1 (OM287082.1), *C. auris* Cauris_II.2 (OM287083.1), *C. auris* B11205 (CP060341.1), *C. auris* Cauris_I.2 (OM287079.1), *C. auris* Cauris_I.3 (OM287080.1), *C. auris* Cauris_I.4 (OM287081.1), *C. auris* MS3054 (KY410388.1), *C. auris* RICU4 (MH124608.1), *C. auris* IV.1 (MW368389.1), *C. auris* IV.2 (MW368392.1), *C. auris* Cauris_IV.9 (OM287086.1), *C. auris* Cauris_IV.10 (OM287087.1), *C. auris* Cauris_IV.11 (OM287088.1), *C. auris* Cauris_IV.12 (OM287089.1), *C. auris* Cauris_IV.13 (OM287090.1), *C. auris* Cauris_IV.14 (OM287091.1), *C. auris* Cauris_IV.7 (OM287084.1), *C. auris* Cauris_IV.8 (OM287085.1), *C. auris* IV.1ERG11F444L (MW368390.1), *C. auris* IV.1TAC1bS611PERG11F444L (MW368391.1), *C. auris* IV.3 (MW368393.1), *C. auris* IV.4 (MW368394.1), *C. auris* IV.5 (MW368395.1), *C. auris* IV.6 (MW368396.1), *C. auris* B11220 (QEO20389.1), *C. auris* B11243 (PSK75255.1), *C. auris* B11245 (QEL61552.1), *C. haemulonii* B11899 (XP_025344294.1).
