# Supplementary material for: Circulation of fluconazole-resistant Candida auris in Peru confirmed by genomic analysis
Source: Mem Inst Oswaldo Cruz. 2026 Jul 31;121:e260027. doi: 10.1590/0074-02760260027 (PMC13426473; doi:10.1590/0074-02760260027)
Supplement: Supplementary material [file 1678-8060-mioc-121-e260027-s1.pdf]

Mem Inst Oswaldo Cruz, Rio de Janeiro, Vol. *121*: e260027, 2026

TABLE

Minimum inhibitory concentration (MIC) values of *Candida auris* isolates against antifungal agents. MIC values are expressed in µg/mL. Blood and tissue samples were classified as infection-related isolates, whereas rectal swabs were considered colonisation isolates

N°	Strain	Sample type	Anidulafungin	Micafungin	Caspofungin	Posaconazole	Voriconazole	Fluconazole	Amphotericin B
1	CA1PER	Blood	0.12	0.25	0.25	0.12	0.25	128	1
2	CA2PER	Blood	0.03	0.06	0.12	0.12	0.5	32	0.5
3	CA3PER	Blood	0.03	0.06	0.12	0.12	0.5	32	1
4	CA4PER	Blood	0.06	0.25	0.06	0.12	0.25	64	0.5
5	CA5PER	Blood	0.06	0.06	0.25	0.06	0.25	32	0.5
6	CA6PER	Rectal swab	0.06	0.12	0.25	0.12	0.12	64	1
7	CA7PER	Blood	0.06	0.12	0.03	0.015	0.12	64	1
8	CA8PER	Blood	0.06	0.12	0.25	0.03	0.5	64	1
9	CA9PER	Rectal swab	0.06	0.12	0.12	0.03	0.25	64	1
10	CA10PER	Rectal swab	0.06	0.12	0.5	0.06	0.25	64	1
11	CA11PER	Rectal swab	0.03	0.06	0.25	0.06	0.12	≥256	0.5
12	CA12PER	Rectal swab	0.06	0.12	0.25	0.03	0.12	128	1
13	CA13PER	Tissue	0.06	0.06	0.12	0.06	0.5	32	0.5
14	CA14PER	Blood	0.06	0.06	0.12	0.12	0.25	32	0.5
15	CA15PER	Blood	2	0.06	0.12	0.12	0.5	128	1
16	CA16PER	Blood	0.06	0.12	0.25	0.015	0.12	64	1
17	CA17PER	Blood	0.06	0.06	0.25	0.12	0.12	64	1
18	CA18PER	Blood	0.06	0.06	0.12	0.03	0.12	≥256	0.5
19	CA19PER	Blood	0.06	0.12	0.25	0.12	0.25	64	1
20	CA20PER	Tissue	0.06	0.12	0.5	0.12	0.25	64	1

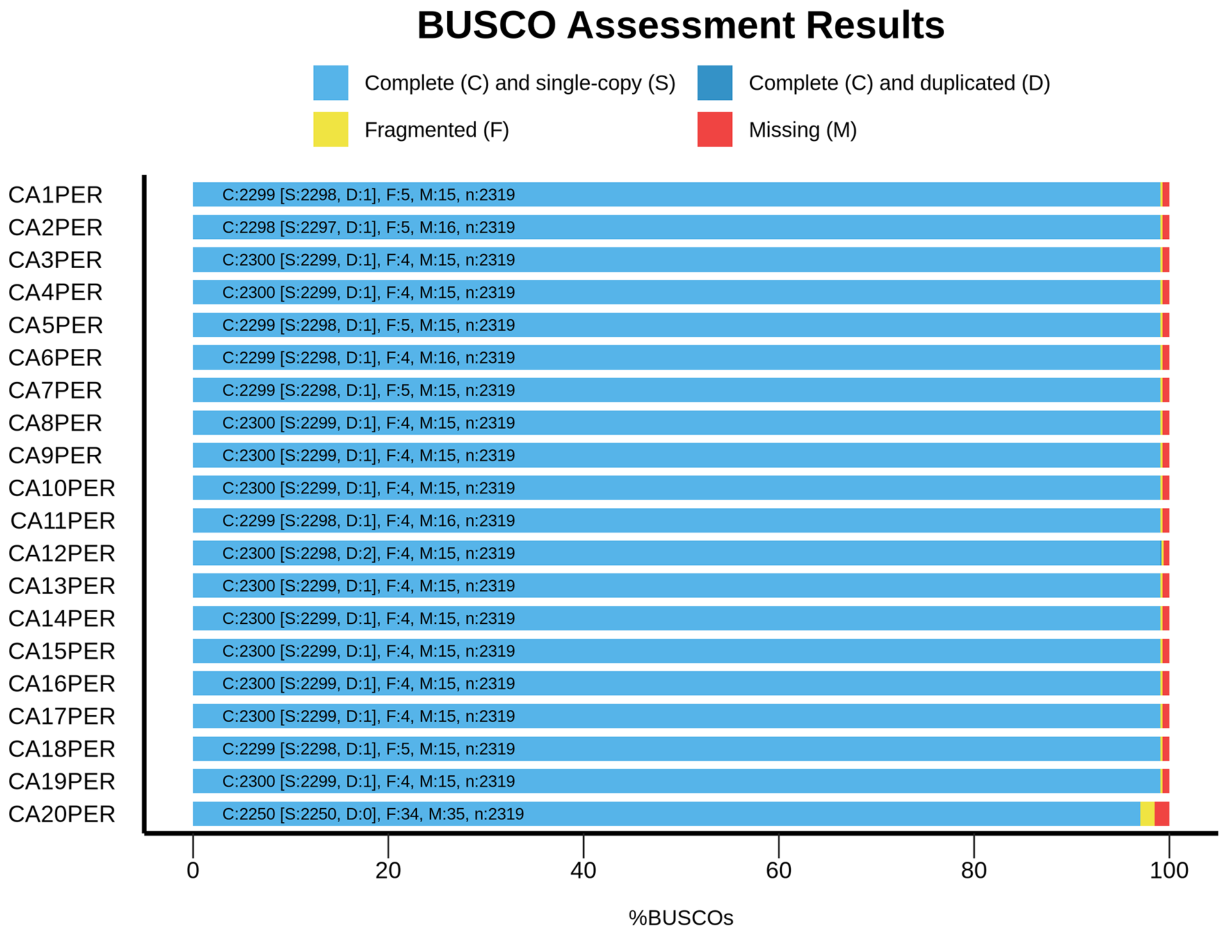

Percentage of universal single-copy orthologous genes identified in the *Candida auris* assemblies using the saccharomycetes_odb12 database (2319 BUSCOs). Light blue bars represent complete and single-copy genes (S); dark blue, complete and duplicated (D); yellow, fragmented (F); and red, missing genes (M)."
