# Supplementary material for: Circulation of fluconazole-resistant Candida auris in Peru confirmed by genomic analysis
Source: Mem Inst Oswaldo Cruz. 2026 Jul 31;121:e260027. doi: 10.1590/0074-02760260027 (PMC13426473; doi:10.1590/0074-02760260027)
Supplement: Peer review history

# Reviewer report: FIRST REVIEW ROUND - REVIEWERS COMMENTS

## REVIEWER #1

Great paper on the molecular epidemiology of C. auris in Peru, highlighting its behaviour in the country and the region. Some minor comments to be considered:

1. Line 38: please re write since its confusing:

Phylogenetic analyses based on whole-genome sequencing (WGS) and single nucleotide 39 polymorphism (SNP) data have identified six geographically distinct clades of Iran), and clade VI (recently proposed and reported in Bangladesh and Singapore) C. auris clades: I (South Asia,), 41 II (East Asia,), III (Africa,), clade IV (South America,), clade V

2. It would be interesting to have access to information about epidemiology of C. auris in Peru. Is it a mandatory notifiable event?

3. Despite all isolates were resistant to Fcz, it will be interesting to see an analysis regarding the differences found in MIC values of isolates associated with colonization and those related to infections

## REVIEWER #2

The manuscript presents a timely and relevant study on the genomic characterization of Candida auris isolates from Lima and Callao, Peru. By integrating antifungal susceptibility testing with whole-genome sequencing, phylogenomic analysis, and molecular docking, the study addresses an important gap in regional genomic surveillance of this emerging multidrug-resistant pathogen.

Overall, the work is aligned with the scope of the journal and has the potential to contribute meaningfully to the field. However, several aspects require moderate to major revision to improve clarity, rigor, and contextualization.

A) The abstract does not adequately convey the significance and novelty of the study. It lacks clear statements about the knowledge gap, the specific objectives, and the work's main contributions. It recommends: 1) Clearly state the research gap (limited genomic data from Peru and South America). 2) Specify the study objectives (e.g., clade assignment, resistance mutation analysis, and genomic epidemiology). 3) Highlight the main findings and their implications for public health and surveillance.

B) The topic is highly relevant, especially considering the global emergence of Candida auris as a priority pathogen. The study provides valuable data from an underrepresented region.

However, the claim of being the "first genomic characterization" requires clarification and stronger support. A recent publication (Cuicapuza et al., 2026) reports on a draft genome from a Peruvian isolate and must be acknowledged. Existing literature must be cited and discussed, including the 2026 report. The novel aspects of this study must be clearly defined (e.g., number of isolates, comparative genomics, integration with phenotypic data, epidemiological insights).

The novelty claim should be reformulated to reflect the actual contribution (e.g., the first multi-isolate genomic analysis or the first integrated genomic-phenotypic study in Peru). Document the gap explicitly by referencing database searches or the lack of prior comprehensive studies.

C) The methodological framework is appropriate and robust, yet it lacks key details necessary for reproducibility.

Please specify: Study design (retrospective or prospective); Sampling timeframe; Inclusion/exclusion criteria; Sampling strategy and rationale for sample size.

line 62: MALDI-TOF, Indicate the database version and whether C. auris spectra were included or updated.

Line 172: Antifungal Susceptibility, Provide MIC distributions in table format (supplementary material recommended).

The results are meticulously arranged and firmly rooted in scientific principles.

Genomic analyses are robust and well presented, the epidemiological data remain largely descriptive. The clarity of the text would be improved by the addition of quantitative presentation.

Discussion: The discussion appropriately links findings to global trends.

The interpretation of ERG11 mutations aligns with existing literature. However, the molecular docking section is overstated. Docking results should be presented as hypothesis-generating, not confirmatory.

Please include a clear limitations paragraph and make the comparison stronger by using regional studies.

D) References are generally appropriate and up to date.

E) Improve clarity and self-sufficiency of figures.

Figure 4 (Line 257): Include embedded legend defining: color codes (e.g., red/gray heatmap), Clade shading (orange), symbols (asterisks, bold), Branch length units

Figure 4C (Line 261): Add a concise legend to panel C (2D interaction map) that defines all symbols (e.g., red semicircles = hydrophobic contacts, dashed lines = H-bonds) and annotate measured distances (Å) for key interactions; explicitly state the 4 Å cutoff used. Improve Table 1 formatting and readability.

Please Correct and clarify the clade/phylogeny paragraph in the Introduction (line 38-41): explicitly list clades I-VI with their geographic associations, remove any garbled manuscriptmanagement text or stray URLs, and cite the primary sources that define clade assignments (including the proposal for clade VI).

Please correct line 47 and 59: change the dot to black.

## AUTHORS' RESPONSE TO THE REVIEWERS

Reply to the reviewer: Thank you very much for your positive assessment of our manuscript. Regarding your comments:

1. Line 38: We appreciate this observation and apologize for the confusion generated by this sentence, which resulted from an error during the formatting/editing process using the citation manager. We have corrected and clarified the paragraph, which now reads: "Phylogenetic studies based on whole-genome sequencing (WGS) and single nucleotide variants (SNPs) have described six geographically distinct clades of C. auris: Clade I (South Asia, mainly India and Pakistan), Clade II (East Asia, primarily detected in Japan and South Korea), Clade III (Africa, predominant in South Africa and other African regions), and Clade IV (South America, mainly reported in Venezuela and Colombia). Clade V has been described based on a limited number of isolates from Iran, while Clade VI is a recently proposed lineage identified in Bangladesh and Singapore." (lines 38-45)

2. Thank you for this comment. We have incorporated information regarding the epidemiological surveillance framework for C. auris in Peru in the Discussion section, clarifying that although notification is mandated through epidemiological alert AE-027-2020, C. auris is not currently included as a formally mandatory notifiable disease under a specific national regulation, but rather managed within the framework of healthcare-associated infection surveillance (lines 348-355).

3. We thank the reviewer for this valuable suggestion. We have compared MIC distributions between isolates obtained from colonization sources (rectal swabs) and those from invasive infections (blood and tissue samples). No relevant differences were observed between these groups, as MIC values remained within comparable ranges across all antifungal agents. This information has been added to the Results section (lines 177-179).

A) The abstract does not adequately convey the significance and novelty of the study.

Reply to the reviewer: We thank the reviewer for this valuable suggestion. The abstract has been revised to clearly state the knowledge gap regarding limited genomic data from Peru and South America, specify the study objectives (epidemiological analysis, clade assignment, phylogenetic reconstruction, and characterisation of antifungal resistance-associated mutations), and highlight the main findings and their implications for public health and genomic surveillance.

B) The topic is highly relevant, especially considering the global emergence of Candida auris as a priority pathogen.

Reply to the reviewer: Thank you for this important observation. The previous claim has been replaced with "the first multi-isolate genomic analysis of C. auris in Peru" throughout the text (Abstract, Introduction and Discussion), to better represent the scope and novelty of our study. We have also incorporated information in the Introduction (lines 51-62) from the first report of C. auris in Peru by Paucar-Miranda et al. (2021) and the recent publication by Cuicapuza et al. (2026). Additionally, both studies are now acknowledged and discussed in the Discussion section. Specifically, we contextualize our findings by confirming that Peruvian isolates belong to clade IV, consistent with previous reports (lines 355-356). We also compare key resistance-associated mutations, such as ERG11 K143R and substitutions (K177R, N335S, E343D), with those reported in Peruvian and other Latin American isolates (lines 371 and 376-378).

The novelty claim should be reformulated to reflect the actual contribution.

Reply to the reviewer: We thank the reviewer for this important comment and the opportunity to clarify the novelty of our study. We have now explicitly stated the existing knowledge gap (lines 348-350), highlighting that genomic data from Peru are limited and mostly based on single-isolate reports. While a recent report described a draft genome from a single Peruvian C. auris isolate, comprehensive genomic analyses across multiple isolates are still lacking. Here, we present the first multi-isolate genomic study of C. auris in Peru, including 20 clinical strains. We further clarify that no previous studies have examined genomic diversity, phylogenetic relationships, and antifungal resistance across multiple isolates in the country. Our study addresses this gap by integrating genomic, epidemiological, and phenotypic data, providing new insights into genomic diversity and fluconazole resistance (lines 395-397).

C) The methodological framework is appropriate and robust, yet it lacks key details necessary for reproducibility.

Reply to the reviewer: We appreciate the reviewer's comment. The Methods section has been revised to clearly specify the study design (retrospective observational), sampling timeframe (2020-2024), inclusion criteria, and sampling strategy, including the rationale for the sample size. These details have been incorporated in the revised manuscript (lines 66-73).

Line 62: MALDI-TOF, Indicate the database version and whether C. auris spectra were included or updated.

Reply to the reviewer: We thank the reviewer for pointing this out. The MALDI Biotyper database (BDAL) used for species identification has now been specified in the Methods section. The acquisition software (FlexControl v3.4) was indicated (lines 78-80).

Line 172: Antifungal Susceptibility, Provide MIC distributions in table format (supplementary material recommended).

Reply to the reviewer: We appreciate the reviewer's suggestion, which helped improve the clarity of the manuscript.

For point 1: We appreciate this suggestion. A detailed table including MIC distributions for all isolates and antifungal agents has now been added as Supplementary Material 1. This information is also referenced in the Results section to improve clarity and data transparency (lines 205-206).

For point 3: The epidemiological data section has been revised to improve clarity by incorporating quantitative information, including percentages and proportions, where appropriate. These modifications complement the descriptive data present in table 1 and enhance the overall readability of the Results section (lines 160-166).

Discussion: The discussion appropriately links findings to global trends.

Reply to the reviewer: We are grateful for the reviewer's careful evaluation.

We re-paraphrase the discussion, in order to present our molecular docking results as a hypothesis-generating way (lines 378-380).

Regarding the limitations of our study, we added several paragraphs (lines 382-394) according to the suggestions.

D) References are generally appropriate and up to date.

Reply to the reviewer: Thank you.

E) Improve clarity and self-sufficiency of figures.

Reply to the reviewer: We appreciate the reviewer's input. We have added an embedded legend in Figure 3 which your description belongs to. Indeed, we did the same with legend of figure 4C, however, we couldn't add the measured distances due to the program LigPlot calculates automatically the type of bonds and shows the plot of interactions unlike Pymol which let us show you specific bonds based on Amstrong distances (Figure 4B). Finally, we have formatted and improved the readability of Table 1, making it clearer and more accessible. Based on your suggestions the dots were changed to black and we clarified the clade/phylogeny paragraph in the introduction.

We are attaching the revised version of the manuscript, with the requested changes highlighted in red and minor revisions made by the authors indicated accordingly. Please let us know if any point requires further clarification.

Best regards,

Msc. Rolando Paredes Gago

Instituto Nacional de Salud (INS) - Perú

Av. Defensores del Morro No. 2268 Chorrillos - Perú

Telephone: (511) 7480000 (annex 1424)

# Reviewer report: REVIEWERS COMMENTS

## REVIEWER #1

The authors correctly addressed the suggestions and comments made.

## REVIEWER #2

No comments.
